# Coverage Path Planning Using Actor–Critic Deep Reinforcement Learning

**DOI:** 10.3390/s25051592

**Published:** 2025-03-05

**Authors:** Sergio Isahí Garrido-Castañeda, Juan Irving Vasquez, Mayra Antonio-Cruz

**Affiliations:** 1 Centro de Innovación y Desarrollo Tecnológico en Cómputo (CIDETEC), Instituto Politécnico Nacional (IPN), Mexico City 07700, Mexico; sgarridoc2000@alumno.ipn.mx; 2 Sección de Estudios de Posgrado e Investigación, Unidad Profesional Interdisciplinaria de Ingeniería y Ciencias Sociales y Administrativas (SEPI-UPIICSA), Instituto Politécnico Nacional (IPN), Mexico City 08400, Mexico; mantonioc@ipn.mx

**Keywords:** coverage path planning, deep reinforcement learning, proximal policy optimization, advantage actor–critic

## Abstract

One of the main capabilities a mobile robot must demonstrate is the ability to explore its environment. The core challenge in exploration lies in planning the route to fully cover the environment. Despite recent advances, this problem remains unsolved. This study proposes an approach to address the coverage path planning problem, where the mobile robot is tasked with exploring and completely covering a terrain using a deep reinforcement learning framework. The environment is divided into cells, with obstacles designated as prohibited areas. The robot is trained using two state-of-the-art reinforcement learning algorithms based on actor–critic methods: Advantage Actor–Critic (A2C) and Proximal Policy Optimization (PPO). By defining a set of observations, states, and a reward function tailored to characteristics of the environment and the desired behavior of the robot, the training process is conducted, resulting in optimized policies for each algorithm. Then, these policies are evaluated to determine the most effective approach to accomplish the proposed task. Our findings demonstrate that actor–critic methods can produce policies capable of guiding a robot to efficiently explore and cover new environments.

## 1. Introduction

Robots are playing an increasingly significant role in the daily lives of humans, taking on tasks ranging from domestic assistance to the automation of complex industrial processes [1]. As technology advances, the ability of these systems to interact autonomously and efficiently with their environment becomes a critical factor for their successful integration into various contexts [2,3]. One of the fundamental skills a mobile robot must possess is the ability to explore and map its surroundings thoroughly and effectively, enabling not only autonomous navigation but also the execution of specific tasks in unknown or dynamic areas.

Coverage Path Planning (CPP) is the task of determining how a mobile robot can achieve the complete observation of each point in an area of interest [4,5]. The strategy aims to maximize efficiency by designing routes that optimize criteria such as time, energy, or the length of the planned path. This objective is achieved through the formulation of algorithms that prioritize minimizing redundant paths, accurate motion calculation, and the avoidance of omissions. In recent years, the development of coverage path planning has gained importance due to its multiple practical applications, such as automated cleaning [6,7], search and rescue [8], dangerous area monitoring [9], and agriculture [10,11], among others.

Advances in deep reinforcement learning algorithms have enabled robots to navigate efficiently around environments with obstacles. The feasibility of the addition of coverage tasks to robot navigation is important for designing systems capable of completely covering an area of interest with obstacles. In this way, reinforcement learning-based coverage path planning could be extrapolated to a variety of applications, resulting in the efficient consumption of different types of resources, the automation of exhausting or complicated tasks due to environmental conditions, or even the replacement of humans in extremely risky jobs.

This paper explores the application of deep reinforcement learning methods, specifically the Proximal Policy Optimization (PPO) [12] and Advantage Actor–Critic (A2C) [13] algorithms, to solve the coverage path planning problem in a discrete cell-based environment. Both algorithms are used to train a mobile robot in a simulated setting, with the goal of enabling it to learn an efficient and complete coverage policy, thereby minimizing coverage time-steps. In addition, a task-oriented reward function is proposed. The proposed approach takes advantage of the actor–critic-based deep reinforcement learning algorithms to adapt to the environment features by abstracting them and making appropriate action decisions at each change in the state according to the objective of obtaining an optimized policy based on the reward function.

The state space, action space, and reward function have been designed, considering a grid environment divided into cells. The design does not rely on the use of a map, which adds a layer of complexity to the problem by requiring the robot to navigate without prior knowledge of the layout. The state space was defined to capture relevant information about the robot’s position, the surrounding environment, and the remaining areas to be covered, while the action space consisted of primitive motion commands. The reward function was structured to incentivize the robot to cover the environment efficiently, penalizing unnecessary movements and collisions, and encourage exploration of uncovered areas.

Experiments were conducted under two conditions: one with no obstacles and the other with fixed obstacles. To evaluate the robustness of the model, 500 experiments were performed in environments with random obstacles, allowing the analysis of the algorithm behavior in more challenging and realistic scenarios. The results obtained not only demonstrate not only the effectiveness of the algorithms in obstacle-free environments, but also their ability to adapt and optimize the coverage path in the presence of obstacles, which represents a significant improvement in the autonomy and efficiency of mobile robots in complex scenarios.

## 2. Related Work

Early work on CPP focused on the discretization of the workspace into a grid and the application of classical search algorithms such as Dijkstra [14,15,16] or A* [17,18,19]. These methods, although simple, were computationally expensive for large environments and did not account for the kinematic constraints of the robots. Cellular decomposition methods, such Morse [20] and Boustrophedon (BCD) [21], divide the area to be covered into a set of obstacle-free cells. The cells are covered by a defined motion pattern, and then the cells overlap with each other, generating transport paths that connect them.

On the other hand, by decomposing the coverage area into a grid, proposed methods define the coverage route by the sequence of primitive movements between adjacent cells. Spiral-STC [22] and variations such as full Spiral-STC [23] and Linked Smooth Spiral-STC [24] implement a sensor-based online path planning methodology that focuses on an established desired movement motion trajectory, using two-dimensional environments composed of uniform cells of similar size to the agent. BA* [25] provides an approach to the solution of a non-map required coverage, using the boustrophedon cellular decomposition motion and A* graph representation in order to solve the problem of a lack of coverage caused by obstacles, using backtracking after completing a path.

With the advancement of computation resources, CPP algorithms based on cell decomposition have been developed to efficiently represent the workspace and find optimal paths. ϵ* [26] proposes an online CPP method using the concept of ETM (Exploration Turing Machine), which acts as a supervisor that guides a robot in the coverage task, taking advantage of Multi-Scale Adaptive Potential Surfaces (MAPSs) dynamically updated with sensor information.

However, these methods are still limited in dynamic environments or with unpredictable obstacles. Learning-based methods help to overcome this limitation, and in particular, reinforcement learning allows us to adapt to specific environmental conditions with multiple coverage approaches, by modeling an appropriate reward function. For known environments, Adaptive Deep Path [27] finds the cell order of the boustrophedon cellular decomposition environment. By using continuous action space, ref. [28] considers a real-world approximation of the nature of the environment adaptable to a mobile robot but also a discrete approximation of environments [29,30], which deals with the limitation of the primitives for accurate movement in grid-based environments.

Reinforcement learning offers an alternative to solve the coverage path planning problem, allowing an agent to learn and adapt strategies through interaction with the environment. Classical methods struggle with scalability, and even those using cell decomposition face the difficulty of dealing with incomplete scenarios, making them less adaptable to unexpected conditions. Actor–critic reinforcement learning methods adapt to unexpected conditions by adjusting their coverage strategy during training. This makes them more flexible, adaptable, and scalable in complex, unknown, or changing environments, overcoming the limitations of traditional methods.

## 3. Background

Reinforcement learning is a technique that allows solving problems where the data are not fixed, providing solutions for a wide range of tasks. One such application is coverage path planning, where the agent does not have complete knowledge of the area to be covered. By leveraging of this partial information with an appropriate reward function, it can be used to train an agent with the capability to solve this problem with an effective generalization due to its ability to adapt to different environment configurations without new training.

Learning-based methods are completely data-dependent. In the case of deep reinforcement learning, the objective is to obtain an optimized policy based on a reward function dependent on the data collected by the agent from the exploration of the environment and the changes that an action performed provokes on it. For this reason, the data used during the training of the agent are not fixed; this type of learning is also known as online.

In deep reinforcement learning, states, actions, and rewards could be defined as an abstraction of the related components in a decision-making process, formalized as the Markov Decision Process (MDP) [31,32]. The set of states, S, is the configuration of the environment, and the finite set A refers to actions that the agent can choose to execute that will result in obtaining a reward belonging to the set R or a reward function. The above process is executed in a time state of the finite set t=0,1,2,..., in which the agent receives the state $S_{t}\in S$ and performs an action $A_{t}\in A$, thus obtaining the pair $\left(S_{t},A_{t}\right)$, and the agent receives the reward $R_{t+1}\in R$; subsequently, it passes to time t+1, where the environment changes to the state $S_{t+1}\in S$ [33].

In policy-based reinforcement learning methods, the agent is instructed to learn a policy based on a probability distribution of actions and to optimize that policy through interaction. On the other hand, the value-based algorithms estimate the value of a state when an action is performed using a value function.

Actor–critic methods are a class of algorithms that combine the strengths of both policy-based and value-based methods. These methods consist of two main components: the actor and the critic. The actor is responsible for selecting actions based on the current policy, mapping states to actions, and updating the policy based on feedback from the critic. In contrast, the critic evaluates the action taken by the actor by estimating a value function, which can be either the state value or action value. This evaluation provides crucial feedback that helps the actor learn which actions are more effective.

The advantage function, in the actor–critic algorithms, measures how much better a specific action is compared to the average action in a given state. This function helps reduce variance in the estimation of policy gradients, consequently achieving more stable updates.

The actor uses policy gradient techniques to adjust its policy, with gradients derived from the value estimates of the critic. This integration of the critic allows for more efficient learning and helps to balance the exploration–exploitation trade-off. The actor encourages exploration by maintaining a stochastic policy, while the critic guides this exploration based on an estimated value, ensuring that the agent not only explores but does so in a way that maximizes learning.

Actor–critic methods also benefit from reduced variance in gradient estimates due to the involvement of the critic, which increases the stability of the learning process. There are various variants of actor–critic methods, including Advantage Actor–Critic (A2C) [13], which uses advantage estimates for updates, and Proximal Policy Optimization (PPO) [12], which is known for its stability in policy updates. Deep Deterministic Policy Gradient (DDPG) [34] is another variant designed for continuous action spaces.

In practice, both the actor and the critic are trained simultaneously. The actor updates its policy based on the critic’s evaluations, leading to improved performance over time. Overall, actor–critic methods are particularly effective in complex environments with large or continuous action spaces, making them a versatile choice for many reinforcement learning tasks. Their design emphasizes sample efficiency and stability, which are crucial for successful training.

### 3.1. Advantage Actor–Critic

In the Advantage Actor–Critic algorithm (A2C) [13], the actor decides which action to perform based on the current state of the agent, while the critic evaluates the action performed, providing an error signal.

The objective is to maximize the expectation of the sum of discounted future rewards; see Equation (1).

(1) $$J\left(\theta \right)=E_{\pi_{\theta }}\left[\sum_{t=0}^{\infty }\gamma^{t}r_{t}\right],$$

where $\pi_{\theta }$ is the parametrized policy by θ, γ is the discount factor, and $r_{t}$ is the reward at time *t*.

The advantage function is responsible for improving the average value of a chosen action compared to all actions in a state. The advantage is given by Equation (2).

(2) A(s,a)=Q(s,a)−V(s),

where Q(s,a) is the action value function and V(s) is the state value function.

The actor loss (Equation (3)) is used to maximize the expected return by adjusting the parameters θ of the policy, while also using the advantage function to evaluate the action performed, comparing it with the average value.

(3) $$L_{\mathrm{actor}}\left(\theta \right)=-E_{\pi_{\theta }}\left[A\left(s,a\right)\log\pi_{\theta }\left(a|s\right)\right]$$

For the critic loss, the MSE between the cumulated reward $R_{t}$ and the estimated value V(s) is used, by adjusting the parameters ϕ, as shown in Equation (4).

(4) $$L_{\mathrm{critic}}\left(\phi \right)=E\left[{\left(R_{t}-V_{\phi }\left(s\right)\right)}^{2}\right]$$

The actor is updated following the gradient policy according to the θ parameters, considering the advantage as shown in Equation (5).

(5) $$\nabla_{\theta }J\left(\theta \right)=E_{\pi_{\theta }}\left[\nabla_{\theta }\log\pi_{\theta }\left(s,a\right)A\left(s,a\right)\right]$$

The critic is updated by minimizing the MSE between the value estimation and the objective reward value as shown in Equation (6).

(6) $$L\left(\phi \right)=E_{\left(s,r,s^{\prime }\right)}\left[{\left(r+\gamma V\left(s^{\prime };\phi \right)-V\left(s;\phi \right)\right)}^{2}\right],$$

where ϕ are the parameters of the neural network that model the critic behavior.

### 3.2. Proximal Policy Optimization

Proximal Policy Optimization (PPO) [12] is an on-policy reinforcement learning algorithm that improves the stability and efficiency of training, by means of an objective function whose goal is maximize the expectation of the sum of discount future rewards; see Equation (1).

PPO uses a loss function (Equation (7)) in order to limit the change in the policy. This helps to maintain the stability of the training.

(7) $$L^{CLIP}\left(\theta \right)=E_{t}\left[\min\left(r_{t}\left(\theta \right)A_{t},\mathrm{clip}\left(r_{t}\left(\theta \right),1-\epsilon ,1+\epsilon \right)A_{t}\right)\right],$$

where $r_{t}\left(\theta \right)$$=\frac{\pi_{\theta }\left(a_{t}|s_{t}\right)}{\pi_{\theta_{\mathrm{old}}},\left(a_{t}|s_{t}\right)}$ is the new-policy-to-old-policy probability ratio, $A_{t}$ is the advantage, and ϵ is the clipping factor.

The actor is updated by optimizing the clipped surrogate objective function, as shown in Equation (8).

(8) $$\nabla_{\theta }J\left(\theta \right)=E_{t}\left[\nabla_{\theta }\min\left(r_{t}\left(\theta \right)A_{t},\mathrm{clip}\left(r_{t}\left(\theta \right),1-\epsilon ,1+\epsilon \right)A_{t}\right)\right]$$

The critic is updated by minimizing the MSE between value estimation and the objective value, as shown in Equation (9).

(9) $$L\left(\phi \right)=E_{t}\left[{\left(r_{t}+\gamma V\left(s_{t+1};\phi \right)-V\left(s_{t};\phi \right)\right)}^{2}\right],$$

where ϕ are critic neural network parameters.

### 3.3. Entropy

In deep reinforcement learning, especially with algorithms like PPO and A2C, the entropy factor is a hyperparameter that encourages exploration by promoting stochasticity in the action distribution. This is a measure of randomness or uncertainty in a probability distribution. In reinforcement learning, higher entropy means a more uniform distribution over actions, which promotes exploration.

By adding an entropy term to the loss function, the algorithms incentivize the policy to explore different actions rather than exploiting known actions that yield higher rewards. This helps prevent the policy from becoming too deterministic too early in training, which can lead to suboptimal policies. The entropy term of the policy is defined as shown in Equation (10).

(10) $$H\left(\pi_{\theta }\left(\cdot |s\right)\right)=-\sum_{a}\pi_{\theta }\left(a|s\right)\log\pi_{\theta }\left(a|s\right)$$

The loss function for PPO includes a term for the entropy of the action distribution. The entropy factor *c* is multiplied by the entropy value and added to the loss function as shown in Equation (11). This encourages the agent to maintain some level of exploration during training.

(11) $$L^{CLIP}\left(\theta \right)=E_{t}\left[\min\left(r_{t}\left(\theta \right)A_{t},\mathrm{clip}\left(r_{t}\left(\theta \right),1-\epsilon ,1+\epsilon \right)A_{t}\right)\right]-c\cdot H\left(\pi_{\theta }\left(\cdot |s\right)\right)$$

A higher entropy factor increases exploration, while a lower value can lead to more exploitation.

Similar to PPO, A2C can incorporate an entropy term in its actor loss function, as shown in Equation (12). The agent is rewarded for maintaining a diverse set of actions. The entropy factor here also balances the trade-off between exploration and exploitation.

(12) $$L_{\mathrm{actor}}\left(\theta \right)=-E_{\pi_{\theta }}\left[A\left(s,a\right)\log\pi_{\theta }\left(a|s\right)\right]-c\cdot H\left(\pi_{\theta }\left(\cdot |s\right)\right)$$

Finding the appropriate value for the entropy factor is important. A high value might lead to excessive exploration and slow convergence, while a low value might cause the agent to settle into suboptimal policies.

## 4. Coverage with Deep Reinforcement Learning

In this paper, coverage path planning is carried out using two deep reinforcement learning algorithms (A2C and PPO). The environment is a two-dimensional grid map, and the agent is modeled as a mobile robot.

In recent years, research related to reinforcement learning has shown that it can be used in various fields. In the case of mobile robotics, it has been used to control robot behavior in order to achieve various goals. One of them is the coverage path planning task, where a robot must be able to cover all the points of an area of interest. To apply deep reinforcement learning algorithms in coverage path planning, it is necessary to take into account the characteristics of the environment and the nature of the actions to be performed. Essentially, for this study, actor–critic algorithms such as A2C and PPO have the characteristic of being able to work with environments in which the agent must adapt to its surroundings and make decisions based on interactions without needing a global map, due to their ability to strike a balance between exploration and exploitation. Also, the action space is defined as discrete in the form of primitive displacements, so the compatibility of this type of discrete action space is a key feature in this specific coverage path planning task—a feature that the A2C and PPO algorithms fulfill.

### 4.1. Environment

The environment is defined as a 2D grid map of size $m\times n\in Z^{+}$ with the coordinate system set as (i,j)∣(i≤m,j≤n), the size of the cell being sufficient to contain the robot inside it. Each cell can be labeled as *uncovered* in the case that the robot has not yet discovered it; *covered* in the case the robot has already discovered it; and *free* in case that the cell is covered and there is no obstacle on it.

The robot is capable of moving over the grid map one cell per movement, in four directions: east, west, north, and south, while it can detect obstacles in adjacent cells. Two scenarios were considered: (i) the robot starts from a valid random position and then starts the coverage task, and (ii) the robot starts from a valid random position, and five obstacles are placed at random positions, and then the robot starts the coverage task, avoiding collisions with the obstacles. The objective in both scenarios is to complete the coverage of the environment, in the fewest number of movements. Once the environment and the robot are defined, the training process is performed to obtain a policy that will serve as the planner.

A valid starting position is defined as a free cell where the robot is able to move at least in one direction over the environment without causing a collision. If the coverage is made without obstacles, all the cell positions are valid; otherwise, at least one of the surrounding cells must be free.

### 4.2. States

A state is the configuration of the environment given the set of information collected by the robot at each time-step. The coverage task implies that the robot is able to know its own position, *q*, in the grid as the coordinates (i,j) (see Equation (13)).

(13) $$q_{t}=\left(i_{t},j_{t}\right)$$

**Figure 1:**
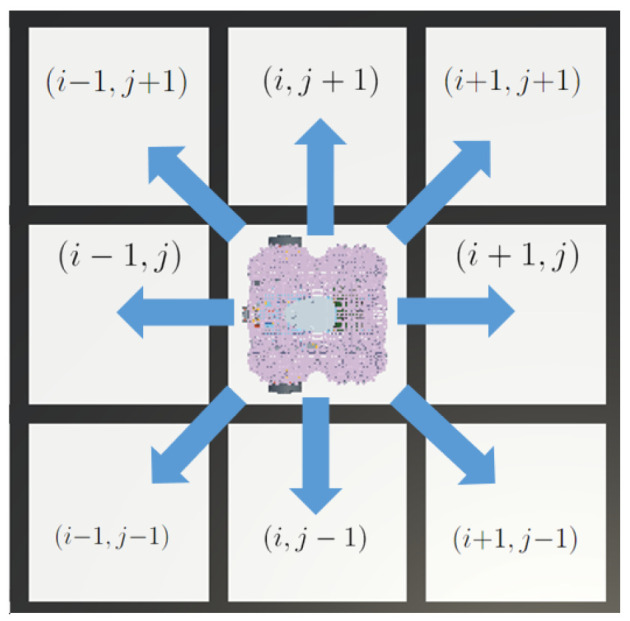
Robot detection capability. Arrows show the direction of the ray and the sensor configuration, allowing the mobile robot to detect the presence of obstacles in the cells around it.

The robot is capable of sensing the environment with eight rays (starting from its center), each one positioned at 45 degrees relative to the previous one, starting from the front of the vehicle. The sensor can detect the presence of obstacles in the next cell in front of it, allowing the robot to detect the eight cells around it. An illustration of the sensor is shown in Figure 1. If the cell is free, then the sensor reads zero. If the cell is occupied by an obstacle, the sensor reads one. The sensor data are allocated into a boolean array of eight elements *z*.

An *uncovered* cell is updated as *covered* depending on the sensor readings of the set of observable cells at the current time-step. Also, only at the beginning of the coverage task, the start position of the robot is also labeled as covered. After that, it is up to the sensors to update the covered cells.

The state is defined in Equation (14).

(14) $$s_{t}=\left\{q_{t},z_{t},c_{t}\right\}$$

where $q_{t}$ is the position coordinates of the robot, $z_{t}$ is the sensor reading, and $c_{t}$ is the number of covered cells.

### 4.3. Actions

The interactions between the robot and the environment induce a change in the state at the time-step *t*, after which an action is performed by the robot, and it will again induce a change in the state at t+1. The mobile robot is able to move through the grid environment cell by movement in the east, west, north, and south directions. Restricting its movement to the four cardinal directions prioritizes simplicity by eliminating complex trajectory planning and geometric calculations. This approach reduces uncertainty in position and orientation, ensuring consistent motion during the coverage task.

Considering the above, the set of discrete actions is defined in Equation (15).

(15) A={east,west,north,south}

With respect to the coordinates *i*, *j* of the robot in the grid, the set of actions is defined in Equation (16).

(16) A(i,j)={(i+1,j),(i−1,j),(i,j+1),(i,j−1)}

**Figure 2:**
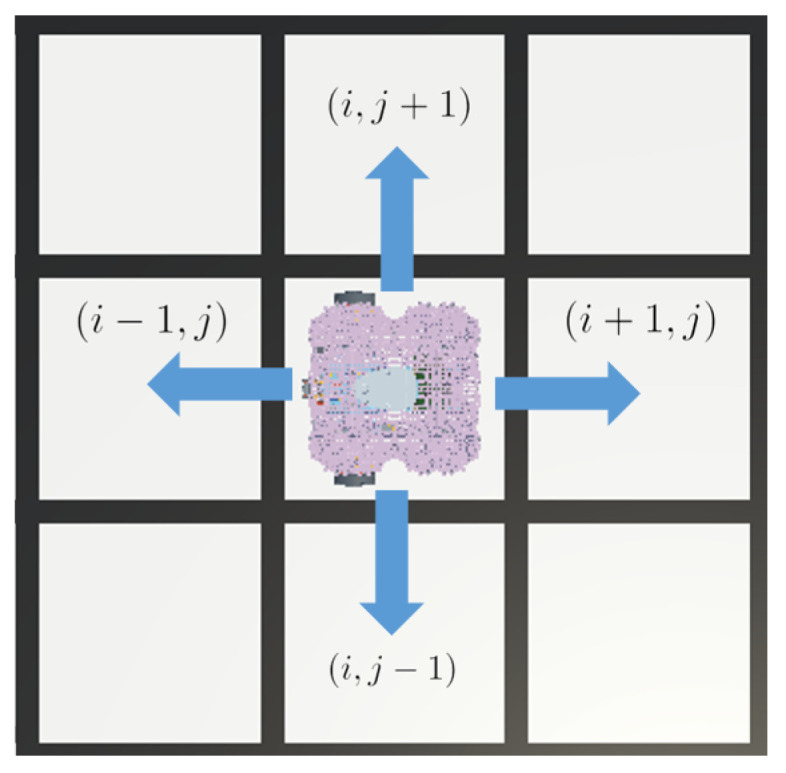
Agent action space. Each mobile robot action changes its a position by one cell in the selected direction.

At each time-step, the robot executes an action of the set, and depending on it, the position of the robot changes at t+1, as shown in Equation (17). The action set is illustrated in Figure 2.

(17) $$q_{t+1}=\left\{\begin{array}{cc} \left(i_{t}+1,j_{t}\right) & \mathrm{if}\;a_{t}=east\\ \left(i_{t}-1,j_{t}\right) & \mathrm{if}\;a_{t}=west\\ \left(i_{t},j_{t}+1\right) & \mathrm{if}\;a_{t}=north\\ \left(i_{t},j_{t}-1\right) & \mathrm{if}\;a_{t}=south \end{array}\right.$$

The robot can only move to a valid position because collisions are not allowed. If the robot chooses an action that would cause it to move out of the coverage area, it will return to the previous position. Therefore, the robot cannot move outside the area of cells to be covered, which completely restricts the positions it can reach.

During training, an episode is defined as the series of steps taken to complete the full coverage of the area of interest, whereas a step corresponds to a change in the state produced by an action. To analyze the results obtained, we observe the mean length of the episode and the mean reward data. Once the training process is performed, a trained model of the policy is obtained, and this model can be loaded to execute the inference in the environment, in order to evaluate the result in episodes with specific configurations.

### 4.4. Transition Function

The valid set of states is represented by the positions that the robot is able to visit, considering the adjacent obstacle position as invalid, which means that the vehicle cannot choose as a next move a cell that contains an obstacle. So, the state is represented as the tuple (q,z,c), resulting in the set of states S as shown in Equation (18).

(18) $$S=\{\left(q,z,c\right)|i\in \left[1,m\right],j\in \left[1,n\right],z\in {\left[0,1\right]}^{8},c\in \left[0,m\times n\right]\},$$

where *m* and *n* are the number of columns and rows of the grid, *z* is the set of sensors, and c is the current number of covered cells.

Once the set of states S is established, the transition function $T(s_{t+1}|s_{t},a_{t}$) is defined considering that the movement of the robot is deterministic according to Equation (15). The definition of the new state depends on the updated robot’s position, the sensor readings, and the addition of the number of the new covered cells resulting from the state transition.

The position at the time-step t+1 depends on the position $q_{t}$ and the action that causes the transition. The update of the position is shown in Equation (19).

(19) $$q_{t+1}=q_{t}+a_{t}$$

Let *M* be the position of the cells that *z* reads with the robot’s position included, and let α be the function that counts the times a cell state changes from *uncovered* to *covered*.

(20) $$c_{t+1}=c_{t}+\alpha \left(M_{t}-M_{t-1}\right)$$

The update of the sensor readings $z_{t+1}$ depends only on the new robot position. The transition function is shown in Equation (21).

(21) $$T\left(\left(q_{t},z_{t},c_{t}\right),a_{t}\right)=\left(q_{t}+a_{t},\;z_{t+1},\;c_{t+1}\right)$$

### 4.5. Reward Function

The reward function is composed of the sum of three independent rewards, based on the desired behavior of the robot. First, $r_{step}$ is intended to motivate the movement of the robot over the environment, and to give it a sense that it must make the coverage in as few movements as possible; therefore, it is necessary to punish the robot at every time-step of the episode, as shown in Equation (22). In order to reward the discovery of “*unknown*” cells, a small reward is given if the number of “*covered*” cells at time *t* is greater than at t−1; see Equation (23). Finally, to reward a route without collisions, a penalty of −10 is given if the previous action caused a collision with an obstacle; otherwise, a small reward is given; see Equation (24).

(22) $$r_{step}=-0.1$$

(23) $$r_{c}=\left\{\begin{array}{c} \left(c_{t}-c_{t-1}\right)\times 0.1\;\;\;\;if\;\;c_{t-1}<c_{t}\\ 0\;otherwise \end{array}\right.$$

(24) $$r_{mov}=\left\{\begin{array}{c} -10\;\;\;\;if\;\;p_{occupied}\\ 0.01\;\;\;\;if\;\;p_{free} \end{array}\right.$$

Equation (25) defines the reward $r_{t}$ at episode *t* as the sum of the three individual rewards.

(25) $$r_{t}=r_{step}+r_{c}+r_{mov}$$

The condition for finishing the episode is that the robot has covered all the cells in the environment. Only in this case, a final reward of 10 is given at the end of the episode.

This reward function guides the robot to balance efficiency in the covering task and safety by avoiding obstacles. A small penalization of −0.1 per time-step encourages the robot to minimize its path length while trying to complete the task quickly. Simultaneously, the positive reward of 0.1 for each newly covered cell incentivizes exploration and ensures coverage. A significant negative reward of −10 for collisions, coupled with episode termination, strongly discourages unsafe actions and forces the robot to learn obstacle avoidance. Finally, a large positive reward of 10 for a complete environment coverage provides a signal for successful task completion, driving the robot to find optimal trajectories. The balance between these rewards guides the robot to learn an efficient and safe coverage policy.

## 5. Experiments and Results

**Figure 3:**
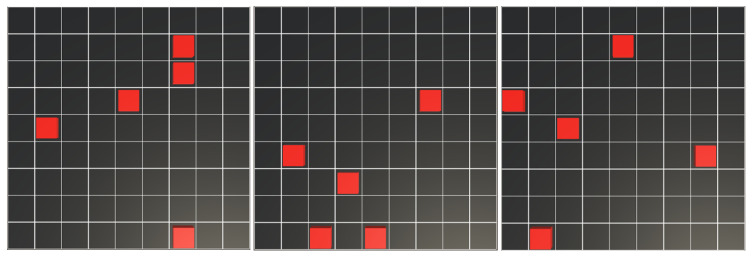
Examples of environment configuration with random obstacles. At the beginning of the episode, the 5 obstacles (red cubes) are placed, each in a different random cell, and the robot’s position will also be in a random cell.

The aim of this study is to train a robot with the A2C (see Algorithm 1) and PPO (see Algorithm 2) algorithms by conducting two experiments for each. The first experiment consists of a task where the robot has to efficiently cover the environment completely, starting from a random position. In the second experiment, five obstacles, each with the same dimensions as the robot, were added to the environment at random positions, centered within individual cells (see Figure 3). The robot has to avoid these obstacles while continuing to perform the coverage task. In each experiment, the set of states, actions, and the reward function are the same as defined in the previous section, with the goal of obtaining the policies that allow the robot to complete the coverage of the environment with the least movements. See Algorithms 1 and 2.
**Algorithm 1** Advantage Actor–Critic (A2C)  1:Initialize policy network $\pi_{\theta }\left(a|s\right)$ and value network $V_{\phi }\left(s\right)$ with random weights θ and ϕ  2:**for** iteration = 1, N **do**  3:   Run policy $\pi_{\theta }$ in environment for *T* steps  4:   **for** t = 1, T **do**  5:     Select action $a_{t}$ according to policy $\pi_{\theta }\left(a_{t}|s_{t}\right)$  6:     Execute action $a_{t}$ and observe reward $r_{t}$ and new state $s_{t+1}$  7:     Store transition $\left(s_{t},a_{t},r_{t},s_{t+1}\right)$  8:   **end for**  9:   **for** t = 1, T **do**10:     ${\hat{A}}_{t}=r_{t}+\gamma V_{\phi }\left(s_{t+1}\right)-V_{\phi }\left(s_{t}\right)$11:     $L_{actor}=-\log\pi_{\theta }\left(a_{t}|s_{t}\right){\hat{A}}_{t}$12:     $L_{critic}={\left(r_{t}+\gamma V_{\phi }\left(s_{t+1}\right)-V_{\phi }\left(s_{t}\right)\right)}^{2}$13:     $L_{entropy}=-\sum_{a}\pi_{\theta }\left(a|s_{t}\right)\log\pi_{\theta }\left(a|s_{t}\right)$14:     $L=L_{actor}+c_{1}L_{critic}-c_{2}L_{entropy}$15:     θ and ϕ16:   **end for**17:**end for**

**Algorithm 2** Proximal Policy Optimization (PPO)
  1:Initialize $\theta_{0}$ and $\phi_{0}$  2:**for** iteration = 1, 2,…, N **do**  3:   Run $\pi_{\theta_{old}}$ for *T* time-steps  4:   $\hat{A}\left(s_{t},a_{t}\right)=\delta_{t}+\left(\gamma \lambda \right)\delta_{t+1}+{\left(\gamma \lambda \right)}^{2}\delta_{t+2}+\ldots$  5:   **for** epoch = 1, 2,…, K **do**  6:     **for** minibatch of size M **do**  7:        $r_{t}\left(\theta \right)=\frac{\pi_{\theta }\left(a_{t}|s_{t}\right)}{\pi_{\theta_{old}}\left(a_{t}|s_{t}\right)}$  8:        $L^{CLIP}\left(\theta \right)={\hat{E}}_{t}\left[\min(r_{t}\left(\theta \right){\hat{A}}_{t},\mathrm{clip}\left(r_{t}\left(\theta \right),1-\epsilon ,1+\epsilon \right){\hat{A}}_{t})\right]$  9:        loss $L\left(\phi \right)={\hat{E}}_{t}\left[{\left(V_{\phi }\left(s_{t}\right)-{\hat{R}}_{t}\right)}^{2}\right]$10:        $L_{entropy}\left(\theta \right)={\hat{E}}_{t}\left[\pi_{\theta }\left(a_{t}|s_{t}\right)\log\pi_{\theta }\left(a_{t}|s_{t}\right)\right]$11:        $\theta \leftarrow \theta +\alpha \nabla_{\theta }\left(L^{CLIP}\left(\theta \right)-c_{1}L\left(\phi \right)+c_{2}L_{entrophy}\left(\theta \right)\right)$12:        $\phi \leftarrow \phi -\beta \nabla_{\phi }L^{VF}\left(\phi \right)$13:     **end for**14:   **end for**15:   Update $\theta_{old}\leftarrow \theta$16:
**end for**



Finally, to test the best policy obtained by the training in both algorithms, the number of obstacles was doubled to observe the behavior of the agent in scenarios not seen during training and also to increase the difficulty of the task of covering the area of interest.

The implementation of these experiments was performed in Unity 3D for the environment and Stable-Baselines3 [35] for the reinforcement learning algorithms. The experiments were run on a Intel Core i7-14700KF processor with an Nvidia RTX 3090 GPU (24 GB VRAM) and 32 GB of RAM, running on Windows 10. Unity version 2021.3.16 and Stable-Baselines3 version 1.0.8 were used.

### 5.1. Training

The training process is divided into two parts: (i) the environment, where the data are collected and (ii) the trainer, which receives the data. The environment is composed of the grid and the mobile robot that will cover the area of interest, equipped with sensors that allow it to detect its surroundings. The area to be covered consists of a grid of m×n where m=n=9 cells in this case. At each time-step, the environment updates the number of cells to cover, depending on the actions taken by the agent. The trainer, which is in charge of executing the reinforcement learning algorithms, through the interaction between the robot and the environment at each time-step, collects a state and sends an action to be performed by the robot, which will produce a new state once it is completed.

Through the training process, the environment and the trainer communicate continuously at each time-step: the environment is initialized with the positions of the elements (robot and obstacles, if applicable), and a state and a reward are collected. The information is sent to the trainer, and it responds with an action to be performed by the robot in the environment, and so on. This process allows learning by interaction with the environment together with the reinforcement learning algorithm. At the end of each experiment, a model with the resulting policy is obtained, representing the strategies learned by the robot to complete the coverage task in both obstacle-free and obstacle environments.

In the first experiment, the robot was trained over 500,000 time-steps; in the second experiment, the training time was extended to the order of millions of time-steps due to the complexity of the coverage task. In both experiments, the maximum episode length was set to 500 time-steps; in the case where this amount was reached, a new episode was started.

**Figure 4:**
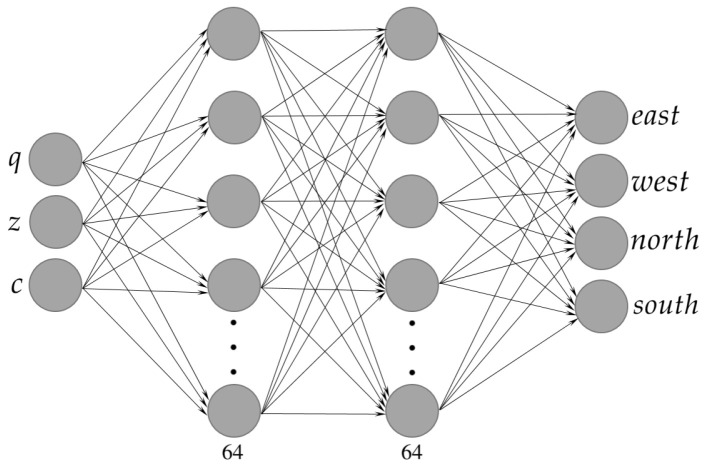
Policy architecture. The policy receives a state and outputs an action. The network’s input consists of the robot’s position *q*; the sensor readings *z*; and the amount of covered cells. Its output is an action from the set A.

The policy architecture is a Multilayer Perceptron (MLP) with two hidden layers, each containing 64 neurons. This MLP takes the state as input and produces an action based on that state; see Figure 4.

The hyperparameter description and values for the A2C and PPO algorithms used during the training process are shown in Table 1 and Table 2. These parameters were obtained empirically by testing small variations to the default parameters of the Stable-Baselines3 [35] implementation.

### 5.2. First Experiment: Robot Random Position

As previously described, this experiment involves the robot starting from a random position in the grid-cell, and then it must cover the whole area of interest. During training, the maximum episode length is 500 time-steps per episode; once this value is reached, a new episode starts.

**Figure 5:**
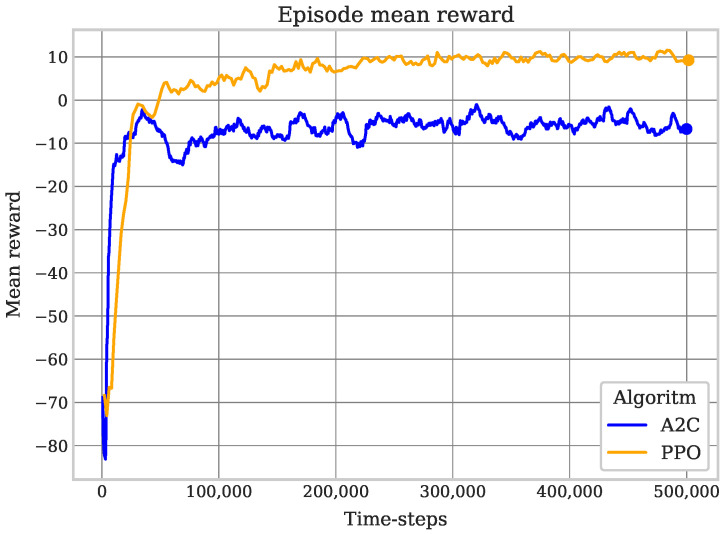
Random PPO vs. A2C mean reward. A larger episode reward means better performance during the coverage task, since the robot’s behavior is closer to that modeled by the reward function.

**Figure 6:**
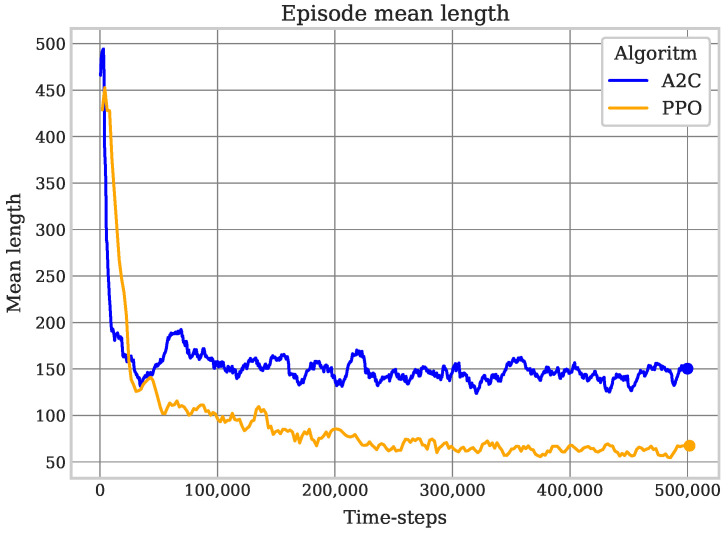
Random PPO vs. A2C episode mean length. A smaller length episode demonstrate a better performance due to fewer movements during the coverage task.

The main indicators that the robot’s training is being carried out as expected are obtaining the values of the mean reward and the mean episode length number of time-steps per episode. The episode mean reward should increase based on the robot’s effectiveness in covering cells in the environment (see Figure 5). On the other hand, the episode mean length is shown in Figure 6 and indicates how fast the robot achieves coverage. The two indicators were measured at three intervals: 100,000, 300,000, and 500,000 training steps, with the last interval also marking the end of the training. It can be seen that PPO was better during the training, since from the first interval, the robot managed to achieve a positive reward, and was in turn also able to increase it in the following intervals. On the other hand, although A2C did not have an erratic performance, it is observed that the metrics behaved unstably during the training. Table 3 shows the results of the training experiment where the starting position of the robot is random.

In order to evaluate the policies resulting from the training, the models corresponding to 100,000, 300,000, and 500,000 time-steps were saved, and 100 episodes were executed in the environment with each model, to obtain data on success and failure in the coverage, as well as the coverage rate, redundancy rate, and the best episode achieved. A scenario was also added where the robot only chooses random positions, in order to compare with both algorithms and to test if the training was indeed effective. The results of this experiment are presented in Table 4.

**Figure 7:**
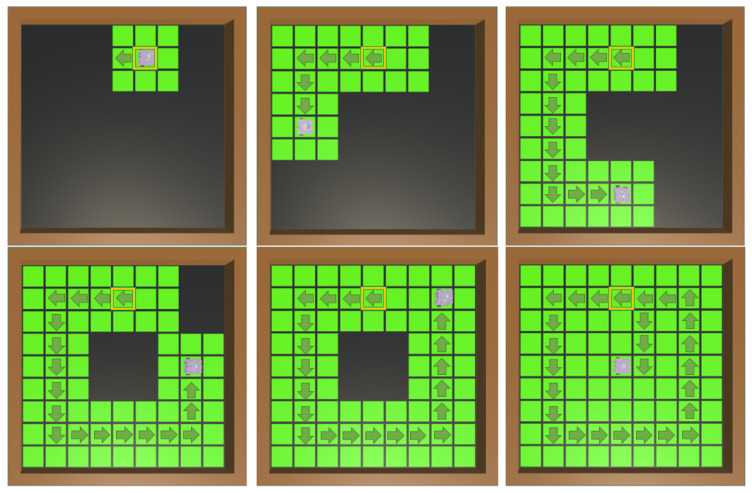
Random position coverage timelapse. The yellow square represents the robot’s starting position in the grid, and the arrows represent its movements. A change in cell color from black to green indicates cell coverage. The timelapse demonstrates the agent’s efficient coverage, minimizing redundant movements.

The best PPO model showed 100% effectiveness with a low level of redundancy; also, this model achieved the best coverage episode. The best A2C resulting model showed a high level of redundancy, although it had a high value of effective coverage. A2C was effective, but in terms of efficiency, PPO showed better results. An example of the coverage process of the best obtained model is shown in Figure 7.

### 5.3. Second Experiment: Environment with Obstacles

In this experiment, the agent begins from a random starting position, and five obstacles are placed at random locations within the grid using a uniform distribution. The experiment was conducted with varying starting positions for both the agent and the obstacles. The position of the agent is set at the beginning of the episode, before the first time-step, and then the obstacle positions are randomly calculated one by one, with the condition that none of the positions can be repeated. If an obstacle’s generated position is the same as a previous one, it is recalculated. After all positions have been determined, the episode begins.

In each episode, the robot must cover the whole area of interest, and in the case where a collision between the robot and an obstacle is produced, a new episode starts, and the robot receives a large penalty (according to the reward function). As in experiment 1, when the episode reaches 500 time-steps without the robot completing the coverage task, a new episode starts.

Table 5 shows data from the training performed in the experiment with the random positioning of the agent and the addition of five obstacles within the environment that must be avoided during the coverage task, at intervals of 10 million, 20 million, and 40 million time-steps.

**Figure 8:**
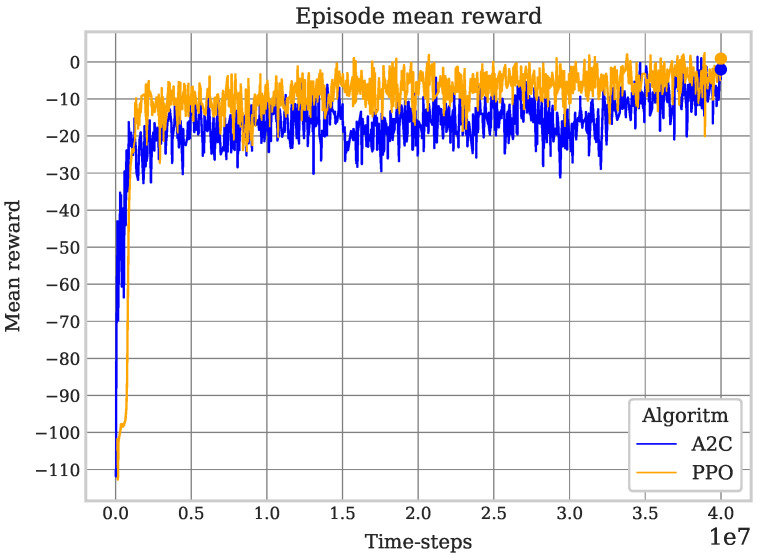
Obstacles PPO vs. A2C mean reward. The obstacle avoidance task requires a greater penalty in the event of a crash compared to redundancy in the covered zones. The training is successful if the reward continues to increase and approaches positive values, even if the mean length does not decrease. As soon as the robot learns to avoid obstacles, it will optimize with lower-value rewards.

**Figure 9:**
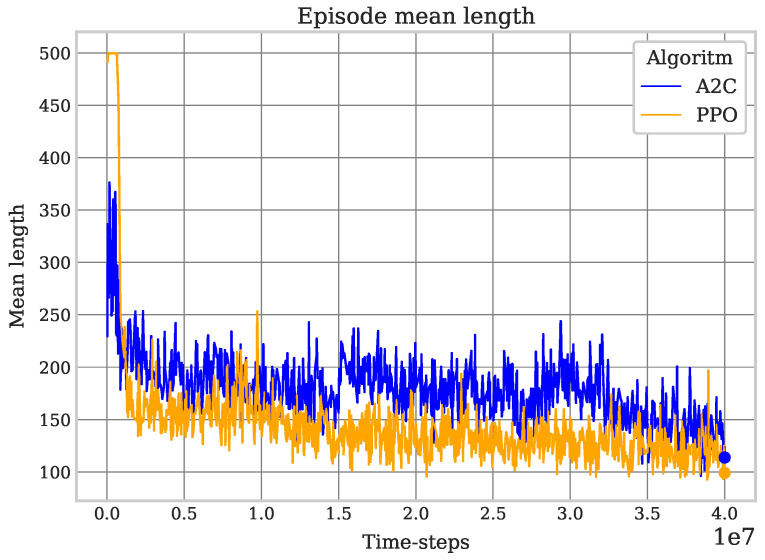
Obstacles PPO vs. A2C episode mean length. Obstacles in the environment imply more complex trajectories for coverage; redundancy in some cases is unavoidable, but the decrease during training is an indicator of the robot’s learning.

Similar to the result of the previous experiment, the performance of PPO is superior to that of A2C. Since this coverage task is more complex, it was necessary to perform a longer training period (on the order of millions of time-steps) in order to obtain consistent values of the mean reward and episode mean length, as well as the expected behavior of the robot corresponding to a successful learning process (avoiding obstacles and completing the coverage). On the other hand, this experiment shows a lack of stability of the A2C algorithm, with lapses in which a deterioration of the robot’s behavior is seen, even though in previous episodes, it seemed to have correctly learned certain patterns that led to a performance corresponding to an expected improvement; with PPO, this does not happen. Due to the randomness of the positions of both the robot and the obstacles, the episode length and the mean reward are susceptible to vary between episodes, but the mean reward increases and the episode mean length decreases in both algorithms during training, demonstrating adequate behavior while ignoring the general performance differences mentioned between the two algorithms; see Figure 8 and Figure 9.

To evaluate the policies resulting from training for this experiment, 100 configurations of agent position and obstacles were selected, in order to evaluate the policy models at 10 M, 20 M, and 40 M time-steps. The same 100 environments were inferred by the policies of both algorithms; the results are shown in Table 6.

**Figure 10:**
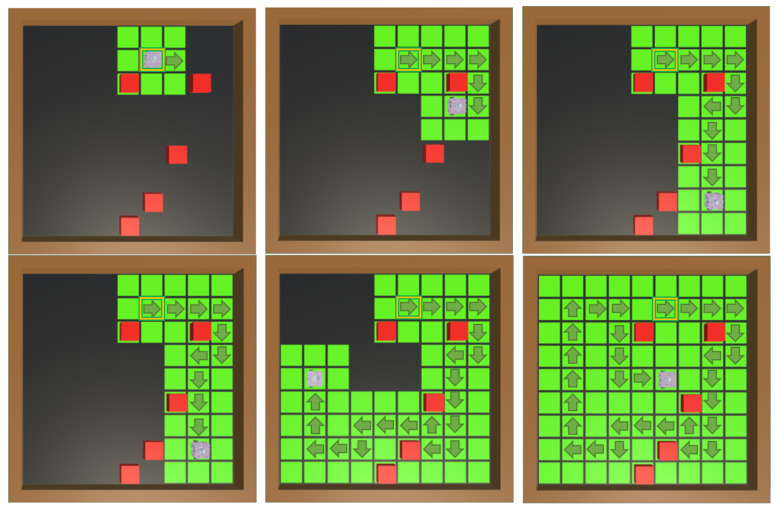
Timelapse of coverage with random positioning of obstacles. The yellow square represents the robot’s starting position in the grid, and the arrows represent its movements. A change in cell color from black to green indicates cell coverage. It can be seen how the agent performs the coverage task while avoiding obstacles (red cubes).

Note that in the case where the agent chooses random actions, colliding with an obstacle is considered a failure; in none of the 100 episodes was complete coverage of the environment achieved. Regarding the algorithms, PPO was again superior to A2C: the 40 M model with PPO managed to complete the coverage task in 99 episodes, achieving a mean episode steps value that was 35.54% better than A2C, not counting the fact that this algorithm still fails to complete the coverage task 3% of the time. The coverage rate is high with both algorithms due to their strong performance in complete coverage success, but PPO showed significantly fewer redundant movements over covered cells compared to A2C, which corresponds to the results of the mean episode steps. An example of the coverage process of the best obtained model is shown in Figure 10.

### 5.4. Policy Test in Double-Obstacle-Density Environments

**Figure 11:**
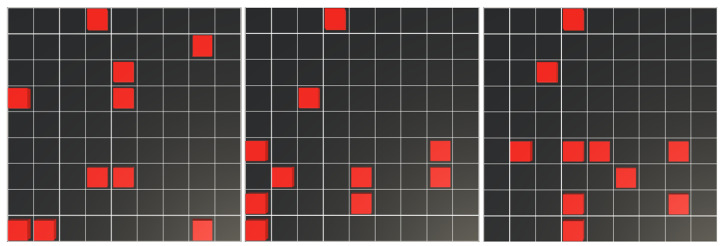
Double-obstacle-density environment configuration. Obstacles are represented as red cubes, and to test the policy, an environment configuration not seen during training is used in 500 episodes.

Considering the results obtained in the previous experiments from training with PPO, a variation of the second experiment was conducted, in which the number of obstacles within the scenario was increased to ten (see Figure 11). In this case, 500 environments with different obstacle configurations of robot and obstacle locations were created. It should be noted that as the density of obstacles in the environment increases, configurations where a set of cells is surrounded by obstacles may arise, making it impossible for the agent to achieve full coverage. These cases were discarded when creating the test environments. In this experiment, the position of the agent is random, as in the previous experiments. The results are shown in Table 7.

**Figure 12:**
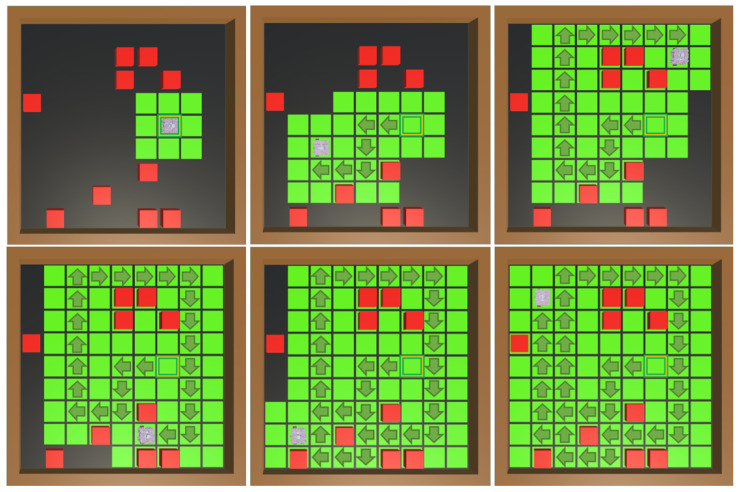
Double-obstacle-density environment complete coverage. The yellow square represents the robot’s starting position in the grid, and the arrows represent its movements. A change in cell color from black to green indicates cell coverage. In environments with many obstacles (red cubes), the policy must perform complex trajectories for the robot to complete the coverage task without crashing.

The results of the PPO policy in these environment configurations demonstrate complete coverage in 91% of the episodes tested. In this variation of experiment 2, it is important to note that the redundancy rate is higher. This is because, during the obstacle avoidance task, the robot often needs to return to a previous position to find a new trajectory. The coverage rate remains high, which means that despite an 8.4% failure of the episodes, in those episodes, near-complete coverage of the environment was achieved. An example of a successful coverage is shown in Figure 12.

### 5.5. Analysis

This study has provided an evaluation of two reinforcement learning algorithms, PPO (Proximal Policy Optimization) and A2C (Advantage Actor–Critic), in the context of coverage path planning in a grid environment divided into cells. By implementing two different scenarios, we were able to analyze how these algorithms perform in both clear environments and environments with obstacles.

In the first scenario, where the agent started from a random position in an obstacle-free space, both algorithms successfully completed the coverage task. However, PPO demonstrated the ability to adapt to the environment and cover the area more efficiently compared to A2C. This adaptability resulted in superior performance in terms of coverage time and trajectory efficiency.

The second scenario, with five obstacles randomly introduced, tested the robustness of the algorithms under complex conditions. While A2C was able to complete the task correctly, PPO again stood out for its stability throughout the training process. Not only did PPO maintain consistent performance, but it also navigated obstacles efficiently, minimizing redundant trajectories and maximizing coverage. In the best-case scenario of both algorithms, although both algorithms completed the coverage, PPO required 18.75% fewer moves than A2C. Additionally, PPO’s redundancy rate was 80.28% lower than A2C, indicating the policy resulting from PPO generated more efficient trajectories.

Also, PPO showed the ability to generalize the behavior of the agent, generating valid coverage trajectories for environments with characteristics not seen during the training (obstacle density doubled), resulting in the accomplishment of a more complex task with a minimal loss of performance.

## 6. Conclusions

The results of the experiments indicate that although A2C is a viable algorithm for coverage path planning, PPO offers some advantages in terms of efficiency, adaptability, and stability, especially in environments with obstacles. This difference in performance could be crucial for robotics applications, where an agent’s ability to react quickly to changes in the environment can determine the success of the task.

This study not only highlights the capabilities of actor–critic reinforcement learning algorithms such as PPO and A2C in coverage planning but also provides a framework for future research to advance the understanding of their effectiveness in complex and dynamic environments. The possibility of increasing the obstacle density in the environment opens up significant implications for autonomous robotics and its application to coverage tasks in real-world scenarios.

In terms of future research, the results of this study suggest that there is great potential to increase the number of obstacles (as seen in the double-density policy test) in a larger environment. This extension not only allows for a more rigorous evaluation of the robustness and adaptability of both algorithms, but also allows for an exploration of the interaction between complexity coverage task and algorithm performance. Such explorations could lead to new optimization strategies and a better understanding of how reinforcement learning algorithms can be applied in real-world situations.

## Figures and Tables

**Table 1:** A2C hyperparameters.

Hyperparameter	Description	Value
Learning rate	The speed at which the policy parameters are updated	7 × $10^{-4}$
Steps per update	Number of time-steps before a policy update	5
Gamma	Determines the importance of future rewards against the immediate reward	0.99
Entropy coefficient	Strikes a balance between exploration and exploitation	0.001
Value function coefficient	Controls the importance of the error in the value network	0.5
GAE lambda	Improves the estimation of the advantage	1
Maximum gradient normalized	Evades overly long gradients that can produce instability	0.5

**Table 2:** PPO hyperparameters.

Hyperpameter	Description	Value
Learning rate	The speed at which the policy parameters are updated	3 × $10^{-4}$
Steps per update	Number of time-steps before a policy update	2048
Batch size	Number of data samples used for the policy update	64
Epoch	Number of policy updates per interaction	10
Gamma	Determines the importance of future rewards	0.99
GAE lambda	Improves the estimation of the advantage	0.95
Clip range	Limits the change in policy per update	0.2
Advantage normalization	Normalization between the difference in the estimated and real values	false
Entropy coefficient	Strikes a balance between exploration and exploitation	0.001
Value function coefficient	Controls the importance of the error in the value network	0.05
Maximum gradient clipping	Limits the gradient to avoid instability	0.5

**Table 3:** Random position training results. A high value of the mean reward indicates a better coverage ratio, and in conjunction with lower values of mean length denotes less redundancy during the coverage process.

Experiment	Time-Steps	Mean Reward	Mean Length
Random	n/a	n/a	n/a
A2C	100,000	−8.47	159.78
	300,000	−7.83	156.02
	500,000	−7.31	149.37
**PPO**	100000	5.24	96.30
	300,000	10.06	70.22
	**500,000**	**10.37**	**67.49**

**Table 4:** Random position test results. The table shows the success rate, episode mean step, coverage rate, redundancy rate, and the best coverage scenario for each model.

Experiment	Time-Steps	Success (%)	Mean Step	Coverage (%)	Redundancy (%)	Best
Random	n/a	57	407.99	82.48	1119.25	155
A2C	100,000	98	188.31	93.82	370.77	40
	200,000	99	168.48	97.53	311.3	36
	500,000	99	157.84	98.77	284.97	40
**PPO**	100,000	100	124.57	100	201.40	36
	200,000	100	85.05	100	104.65	34
	**500,000**	**100**	**62.84**	**100**	**46.13**	**28**

**Table 5:** Training results of environment with obstacles. The improvement in the robot is consistent across both algorithms due to the increase in the mean reward and the decrease in the mean length during training.

Experiment	Time-Steps	Mean Reward	Mean Length
Random	n/a	n/a	n/a
A2C	$10\times 10^{6}$	−13.55	189.71
	$20\times 10^{6}$	−17.48	181.17
	$40\times 10^{6}$	−2.02	115.75
**PPO**	$10\times 10^{6}$	−5.08	134.56
	$20\times 10^{6}$	−7.90	128.64
	$40\times 10^{6}$	**0.87**	**99.03**

**Table 6:** Test results of environment with obstacles. The table shows the success rate, episode mean step, coverage rate, redundancy rate, and the best coverage scenario for each model.

Experiment	Time-Steps	Success (%)	Mean Step	Coverage (%)	Redundancy (%)	Best
Random	n/a	0	500	-	-	
A2C	$10\times 10^{6}$	76	330.39	95.33	334.72	89
	$20\times 10^{6}$	78	448.30	95.81	489.47	75
	$40\times 10^{6}$	97	162.55	99.37	113.88	48
**PPO**	$10\times 10^{6}$	95	198.14	99.26	160.71	47
	$20\times 10^{6}$	97	167.66	99.96	120.6	44
	$40\times 10^{6}$	**99**	**104.78**	**99.99**	**37.86**	**39**

**Table 7:** Results of the double-obstacle-density experiment. To validate the obtained policy, a new scenario is proposed, where the number of obstacles is doubled. The policy must generalize between environments with different obstacles dispositions, in consequence accomplishing the coverage task.

Episodes	Success (%)	Mean Steps	Coverage (%)	Redundancy (%)	Best
500	91.6	234.63	98.85	169.68	49

## Data Availability

Data are available on request.
